# CaReMe-CKD-HF–Epidemiology of Heart Failure in Chronic Kidney Disease: A Retrospective Analysis of Routine Administrative Data from a German Hospital Network

**DOI:** 10.3390/jcdd12110448

**Published:** 2025-11-19

**Authors:** Lars Stellmacher, Sebastian König, Johannes Leiner, Vincent Pellissier, Sven Hohenstein, Jakob Birnbaum, Stefan Kwast, Carolin Schanner, Ralf Kuhlen, Andreas Bollmann

**Affiliations:** 1 Department of Electrophysiology, Heart Center Leipzig at University of Leipzig, 04289 Leipzig, Germany; lars.stellmacher@medizin.uni-leipzig.de (L.S.);; 2 Helios Health Institute, Real World Evidence and Health Technology Assessment, 13125 Berlin, Germany; 3 Department of Visceral, Transplant, Thoracic and Vascular Surgery, University Hospital Leipzig, 04103 Leipzig, Germany; 4 AstraZeneca, Medical Affairs, 22763 Hamburg, Germany; 5 Helios Health, 13125 Berlin, Germany

**Keywords:** chronic kidney disease, heart failure, epidemiology, administrative data, in-hospital mortality, hospital readmissions, time-to-event rates

## Abstract

Background: Heart failure (HF) is a frequent and clinically relevant comorbidity in patients with chronic kidney disease (CKD). Both conditions are closely linked through hemodynamic, neurohormonal, and inflammatory mechanisms, resulting in excess morbidity and mortality. However, nationwide epidemiological data for hospitalized patients with CKD in Germany are scarce. This study aimed to determine the prevalence and outcomes of HF in CKD using a large real-world dataset. Materials and methods: We conducted a retrospective, observational study analyzing administrative data from 87 German hospitals between 2016 and 2022 (*n* = 48,011 CKD index cases). Patients were stratified into CKD with HF (*n* = 22,085; 46%) and CKD without HF (*n* = 25,926; 54%). Additional electronic medical records (EMR) from 23,377 CKD patients of the Heart Center Leipzig were included. All hospital readmissions were considered during the follow-up period. Comparative statistics are presented for each group. Results: CKD patients with HF were older (76.5 ± 10.8 vs. 71.5 ± 14.3 years, *p* < 0.001), more frequently affected by cardiovascular and metabolic comorbidities, and had worse renal function. In-hospital mortality was higher in CKD-HF than CKD-no-HF patients (16.5% vs. 6.7%; odds ratio [OR] 2.73, 95% CI 2.58–2.89; *p* < 0.001). During follow-up, readmissions occurred earlier (175 ± 266 vs. 212 ± 309 days, *p* < 0.001) and more frequently in the CKD-HF group (20.3 vs. 17.7 events per 100 patient–years). Follow-up in-hospital mortality was also increased in CKD-HF patients (13.3% vs. 8.7%; OR 1.62, 95% CI 1.52–1.73; *p* < 0.001). Discussion: In this large, multicenter, real-world cohort, HF was highly prevalent in CKD and associated with substantially increased morbidity and mortality. These findings highlight the considerable public health burden of HF in CKD and the urgent need for targeted prevention and management strategies.

## 1. Introduction

Chronic kidney disease (CKD), an irreversible and progressive condition, predominantly affects people of advanced age [1,2] and has a worldwide prevalence of 9.1–13.4% in the general population [1,3,4]. The condition is a major contributor to morbidity and mortality in affected patients, increasing hospitalization and death rates as well as exerting a substantial socioeconomic impact [5,6,7,8]. CKD shares etiological aspects and risk factors with other common diseases, such as type 2 diabetes mellitus [9,10,11] and various cardiovascular diseases (CVDs) [12,13,14,15,16]. In patients with both CKD and CVD, the diseases can negatively affect each other [17,18,19].

One of the most common cardiovascular comorbidities in CKD is heart failure (HF) [20,21]. The bidirectional relationship between CKD and HF is mediated by multiple mechanisms. CKD promotes HF through volume overload, hypertension, anemia, activation of the renin–angiotensin–aldosterone system, sympathetic overactivity, systemic inflammation, and endothelial dysfunction [14,16,17,20]. Conversely, HF accelerates CKD progression by impaired renal perfusion, increased venous congestion, and repeated exposure to nephrotoxic agents, a constellation summarized as the “cardiorenal syndrome” [17,20].

Recent epidemiological studies underscore the clinical importance of this relationship.

Bansal et al. reported absolute HF incidence rates of 22.0 per 1000 person–years in CKD patients [21]. Data from the United States Renal Data System (USRDS) reported prevalence rates of ~28% in CKD populations [22,23]. The German Chronic Kidney Disease (GCKD) study found an HF prevalence of 43% in patients with moderate CKD [24]. The international CaReMe CKD study observed a pooled prevalence of 24.5% across 11 countries, with higher rates in the German subcohort (~39%) [1]. These findings highlight both the variability of results depending on study design and population, and the consistently high burden of HF in CKD.

In parallel, treatment options for patients with CKD and HF have changed considerably in recent years. Sodium–glucose cotransporter 2 (SGLT2) inhibitors, such as dapagliflozin and empagliflozin, significantly reduce renal disease progression, HF hospitalizations, and mortality in patients with and without diabetes [25,26,27]. Additional agents, such as non-steroidal mineralocorticoid receptor antagonists (finerenone) and angiotensin receptor–neprilysin inhibitors, further expand therapeutic perspectives [26]. These advances underline the urgent need for updated epidemiological data to inform the implementation of new treatment strategies in real-world populations.

Despite these developments, specific evidence on HF prevalence and outcomes in CKD patients in Germany remains limited. Most available studies have either focused on selected cohorts or provided pooled international data. Nationwide analyses of hospitalized CKD patients are lacking, although such data may better reflect the burden of disease encountered in clinical practice.

We therefore conducted the CaReMe-CKD-HF study as a German substudy of the international CaReMe initiative, a study initiative for research of CArdiovascular-REnal-MEtabolic diseases. We hypothesized that HF is highly prevalent in this population and associated with worse outcomes. Our study aimed to characterize CKD patients with and without HF, investigate prevalence, comorbidities, hospital readmissions, and mortality rates, and thereby provide up-to-date, real-world epidemiological data for the German healthcare system.

Heart failure is a common and clinically relevant comorbidity in chronic kidney disease, with prior studies such as CaReMe [1], USRDS [22,23] and GCKD [24] reporting variable prevalence and outcome data. However, robust nationwide evidence on HF in hospitalized CKD patients in Germany is lacking, particularly regarding prevalence, mortality, and readmission outcomes. The present study addresses this gap by analyzing >48,000 CKD cases from 87 hospitals, thereby providing real-world data with direct implications for risk stratification, integrated management, and healthcare planning. This study was conducted with AstraZeneca as the sponsor.

## 2. Materials and Methods

### 2.1. Study Design and Setting

This retrospective, observational study was based on two complementary data sources: (1) administrative claims data from 87 German Helios hospitals between 1 January 2016 and 2 August 2022, and (2) electronic medical records (EMR) from the Heart Center Leipzig during the same period. The Helios hospital network is the largest private hospital provider in Germany, covering a broad range of tertiary and secondary care.

### 2.2. Population, Sample Size and Inclusion

**Figure 1:**
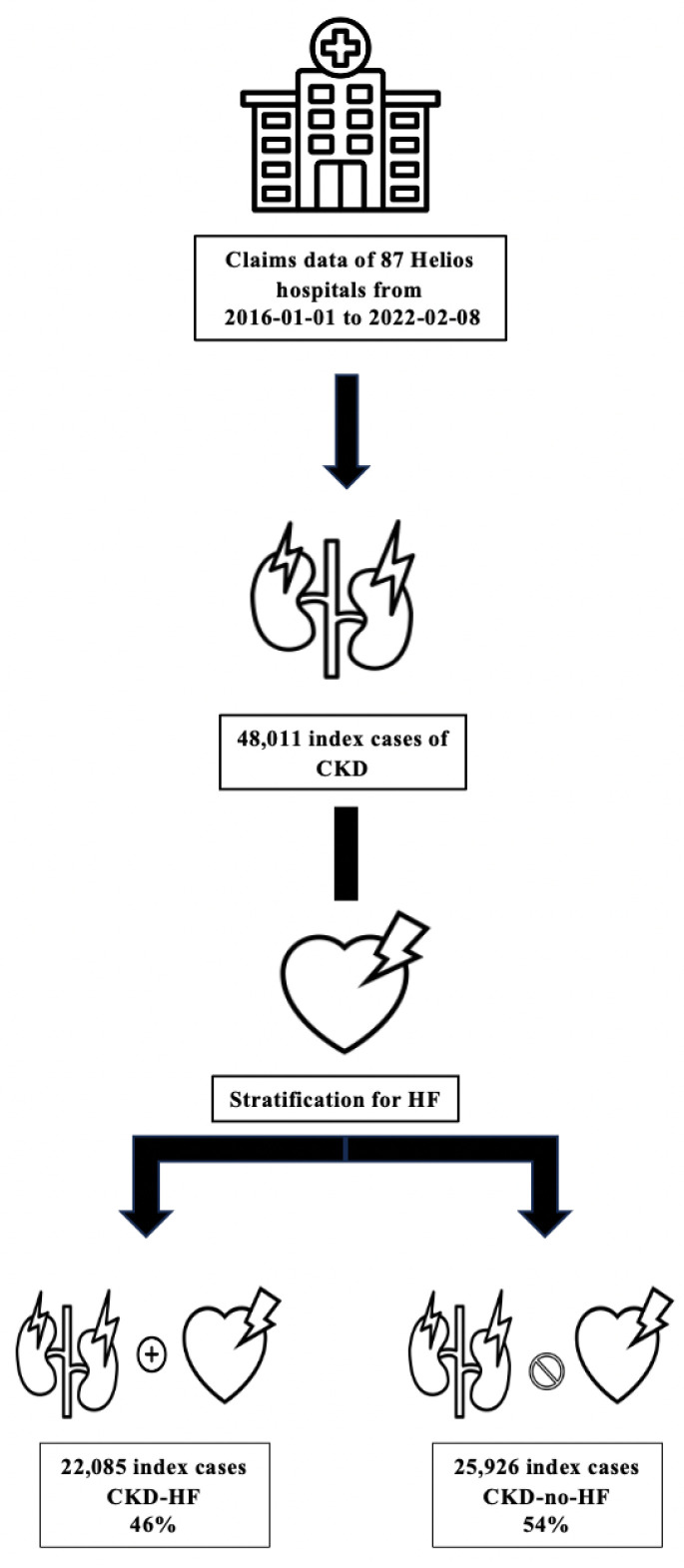
Stratification flow chart. Overview of a hospitalized chronic kidney disease patient selection and stratification according to heart failure status. Abbreviations: CKD—chronic kidney disease; HF—heart failure.

All hospitalized patients aged ≥18 years with a diagnosis of CKD were eligible. Individual index cases were defined as the first hospital stay during the observation period that fulfilled prespecified criteria for CKD according to ICD-10 and/or OPS codes. In the claims cohort, 48,011 index cases were identified. The EMR cohort included 23,377 CKD patients treated at the Heart Center Leipzig. Given the very large sample size, no formal power calculation was performed. All eligible cases were included, ensuring maximal coverage and external validity. Patients with type 1 diabetes were excluded, while patients with type 2 diabetes were included. An overview of the patient selection and stratification process is shown in Figure 1.

### 2.3. Data Collection Tools and Procedures

In the claims cohort, diagnoses were identified using ICD-10-GM codes, and procedures by OPS codes (German adaptation of the WHO International Classification of Procedures in Medicine). CKD stages and comorbidities were encoded within the index case; in case of multiple entries, the more severe CKD stage was recorded.

In the EMR cohort, CKD was defined using the estimated glomerular filtration rate (eGFR, CKD-EPI equation) and supplemented by laboratory parameters and echocardiographic data. The first available value within the index case was selected if multiple laboratory values were present. All ICD-10 and OPS codes used for case definition, comorbidities, and procedures are provided in Supplemental Tables S1 and S2.

### 2.4. Variables and Definitions

CKD stage was defined according to ICD-10 coding in claims data and by eGFR in EMR data. Heart failure (HF) was identified by ICD-10 codes in both datasets. In the EMR cohort, echocardiography allowed stratification into HF with reduced (HFrEF), mildly reduced (HFmrEF), and preserved ejection fraction (HFpEF). Acute kidney injury (AKI) was identified by ICD-10 encoding. The primary outcomes were in-hospital mortality during the index stay and during follow-up, as well as hospital readmissions (all-cause, emergency, and elective). Secondary outcomes included time to first readmission, cause-specific readmissions, and event rates per 100 patient–years. Deaths were further categorized into renal and cardiovascular causes, based on main discharge diagnoses.

### 2.5. Ethical Considerations

The study was approved by the ethics committee of the University of Leipzig (ID: 010/21-ek) and the Helios Kliniken GmbH data protection authority. Patient data were processed in double pseudonymized form, with no access to identifiable information. Informed consent was not required due to the retrospective design. The study followed the STROBE recommendations for observational analyses.

### 2.6. Statistical Analysis

Comparisons between CKD patients with and without HF were performed. Dichotomous variables were analyzed using Fisher’s exact test, continuous variables with Student’s *t*-test. Ordinal and categorical variables are presented as proportions, continuous variables as mean ± standard deviation for normally distributed data or median [IQR] for skewed data. *p*-values < 0.05 were considered statistically significant. Odds ratios (ORs) with 95% confidence intervals (CIs) were calculated where appropriate. Follow-up analyses were based on all readmissions after the index case, including time-to-first-event, cumulative event rates, and event rates per 100 patient–years. Patients who died during the index stay were excluded from follow-up analyses. All statistical analyses were performed using R software (version 4.0.2; R Foundation for Statistical Computing, Vienna, Austria).

### 2.7. Missing Data

In the claims cohort, no data were missing. In the EMR cohort, NT-proBNP values and some laboratory parameters were missing in a substantial proportion of patients. Missing values are summarized in Supplemental Table S3. No imputations were performed.

## 3. Results

### 3.1. Characterization of the Index Population

In the claims cohort, 48,011 CKD index cases were identified; 22,085 (46%) had concomitant HF. The overall mean age was 73.8 ± 13.1 years. Patients with HF were older than those without HF (76.5 ± 10.8 vs. 71.5 ± 14.3 years, *p* < 0.001). The proportion of female patients was slightly lower in the HF group (42.9% vs. 44.9%, *p* < 0.001).

CKD stage 5 was most frequently recorded overall (37.6%). Distribution differed by HF status, with stage 5 less common in CKD-HF than in CKD-no-HF (32.3% vs. 42.2%, *p* < 0.001, Table 1). CKD-HF patients more often required dialysis (44.2% vs. 38.9%, *p* < 0.001) and less often had kidney transplantation (1.0% vs. 1.8%, *p* < 0.001). Acute kidney injury (AKI) occurred in 56.4% of all patients and was more frequent in CKD-HF than in CKD-no-HF (66.6% vs. 47.8%, *p* < 0.001).

Encoded CKD etiologies included primary glomerular nephropathy (34.2%), diabetic nephropathy (33.1%), hypertensive nephropathy (15.4%), and tubule–interstitial disease (5.5%). Comorbidities also showed group differences: hypertension, coronary artery disease, stroke, type 2 diabetes, hyperlipidemia, and chronic obstructive pulmonary disease were more frequent in CKD-HF, while malignancy was more frequent in CKD-no-HF.

In the EMR cohort (*n* = 23,377), 69% of CKD patients also had HF. The overall mean age was 74.5 ± 9.9 years. Kidney function was lower in CKD-HF compared with CKD-no-HF (mean eGFR 50.4 ± 19.4 vs. 56.9 ± 19.2 mL/min/1.73 m^2^, *p* < 0.001). Among CKD-HF patients with echocardiographic data, 39.4% had HFrEF, 24.7% HFmrEF, and 35.9% HFpEF. Impaired LVEF was recorded in 7.4% of CKD-no-HF patients (see Table 2).

### 3.2. Mortality at Index In-Hospital Stay

In the claims cohort, overall in-hospital mortality at index stay was 11.2%. Renal causes accounted for 7.1% of deaths, cardiovascular causes for 1.9%. Mortality was higher in CKD-HF compared with CKD-no-HF (16.5% vs. 6.7%, *p* < 0.001, Table 3).

In the EMR cohort, index in-hospital mortality was lower overall. Mortality was again higher in CKD-HF than in CKD-no-HF (2.1% vs. 0.6%, *p* < 0.001, Table 3).

### 3.3. Follow-Up Analysis

In the claims cohort, 50.1% (*n* = 24,046) of index patients were readmitted during follow-up. Readmission was slightly less frequent in CKD-HF compared with CKD-no-HF (49.1% vs. 50.9%, *p* < 0.001). Mean time to readmission was 196 ± 291 days; patients with HF were readmitted earlier than those without HF (175 ± 266 vs. 212 ± 309 days, *p* < 0.001, Table 4).

Among readmitted patients, 10.8% died during follow-up, at a mean of 439 ± 468 days after the index case. Mortality was higher in CKD-HF compared with CKD-no-HF (13.3% vs. 8.7%, *p* < 0.001) and occurred earlier (414 ± 447 vs. 471 ± 492 days, *p* < 0.001, Table 5). Emergency readmissions were more frequent in CKD-HF (38.2% vs. 36.2%, *p* < 0.001), with higher mortality during emergency readmissions (10.4% vs. 6.5%, *p* < 0.001).

**Figure 2:**
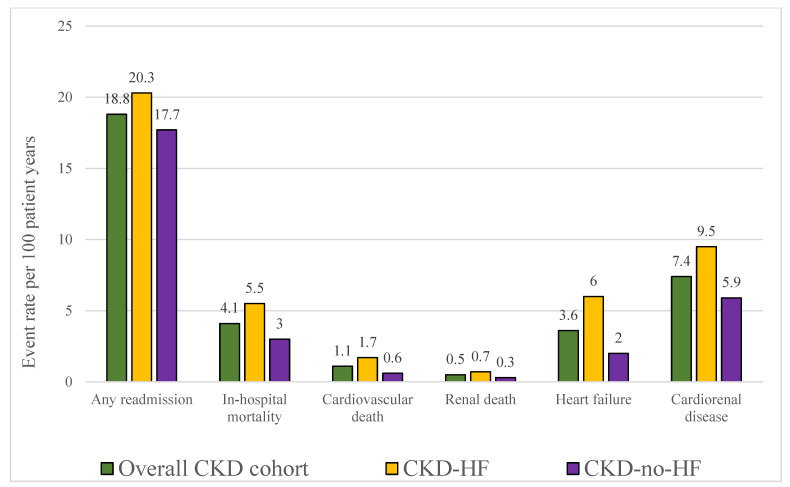
Index population event rates per 100 patient–years, displayed for the overall chronic kidney disease (CKD) cohort and stratified by heart failure (HF) status. Bars represent rates of any readmission, in-hospital mortality, cardiovascular death, renal death, heart failure and cardiorenal disease. Abbreviations: CKD—chronic kidney disease; HF—heart failure.

Event rates per 100 patient–years were 18.8 for all-cause readmissions in the total cohort, higher in CKD-HF than in CKD-no-HF (20.3 vs. 17.7). The most frequent readmission causes were ESRD/dialysis (7.0 per 100 patient–years), kidney disease (4.7 per 100 patient–years), and HF (3.6 per 100 patient–years). In the CKD-no-HF group, HF-related readmissions occurred at 2.0 events per 100 patient–years (Figure 2).

In the EMR cohort, 25.4% of patients (*n* = 5948) were readmitted. Mean time to readmission was 313 ± 401 days, shorter in CKD-HF than in CKD-no-HF (293 ± 387 vs. 365 ± 431 days, *p* < 0.001). Follow-up in-hospital mortality was higher in CKD-HF compared with CKD-no-HF (2.1% vs. 0.6%, *p* < 0.001).

## 4. Discussion

In this large, nationwide analysis of more than 48,000 hospitalized CKD patients, HF was present in almost half of all cases and was associated with increased comorbidity, excess in-hospital mortality, and earlier and more frequent readmissions during follow-up. These findings extend previous epidemiological data and underscore the major public health burden arising from the coexistence of CKD and HF in Germany.

Our prevalence estimates are higher than those reported in many international cohorts. The global CaReMe-CKD study, which pooled data from 11 countries, observed a prevalence of 24.5% for HF among CKD patients, and the German subcohort reported 39% [1]. The GCKD study found a prevalence of 43% in patients with moderate CKD, using Gothenburg criteria for HF [24]. In contrast, the USRDS reports indicated rates around 28% in 2016 and 2020 [22,23]. The ARIC study reported an incidence of 17.7 per 1000 patient–years for individuals with reduced eGFR but no baseline HF [28]. Differences in case definitions, inclusion criteria, and data sources likely explain the higher prevalence in our cohort. Our approach, based on ICD/OPS coding, resembles the “diagnosed CKD” definition in CaReMe, which consistently yielded higher HF rates than “measured CKD” cohorts [1].

Beyond prevalence, our study provides novel insights into outcomes. In-hospital mortality was 16.5% for CKD patients with HF, more than twice the rate of CKD patients without HF (6.7%). Chen et al. recently developed and validated a nomogram to predict in-hospital mortality in patients with congestive HF and CKD, underscoring the high short-term risk in this population and supporting the prognostic relevance of our observations [29]. Follow-up analyses revealed that CKD-HF patients were not only more likely to die (13.3% vs. 8.7%) but also died earlier (414 vs. 471 days after index case). These findings confirm HF as a major driver of adverse prognosis in CKD, adding time-to-event information that has been underreported in prior studies. We also observed that emergency readmissions were more frequent and more lethal in the HF group, further highlighting the instability of this patient population.

The pathophysiological links between CKD and HF provide a biological rationale for these findings. CKD promotes HF through sodium and water retention, activation of the renin–angiotensin–aldosterone system, sympathetic overactivity, anemia, and systemic inflammation, all contributing to adverse cardiac remodeling and impaired function [14,16,17,20]. Conversely, impaired cardiac output in HF reduces renal perfusion, perpetuating a cycle of worsening kidney function. The frequent occurrence of AKI in our CKD-HF cohort (66.6% vs. 47.8% in CKD-no-HF) may reflect this bidirectional interplay and likely contributes to the higher mortality observed.

From a clinical perspective, our results emphasize the need for systematic HF screening in CKD, particularly in advanced stages where prevalence and risk are highest. Integration of echocardiographic and biomarker-based assessment into routine care could help identify high-risk patients [30,31]. Our data also support the implementation of integrated nephrology–cardiology care pathways to reduce emergency readmissions and improve long-term outcomes. Therapeutically, SGLT2 inhibitors have demonstrated consistent benefits across CKD and HF populations. In DAPA-CKD, dapagliflozin reduced the primary kidney–cardiovascular composite by about 39% (HR ≈ 0.61) and improved survival, with benefits observed in patients with and without HF as well as with and without diabetes [25,27]. Contemporary reviews corroborate renal and HF benefits and support earlier, broader implementation in multimorbid CKD–HF patients [26]. Wider adoption of such therapies in Germany may help to reduce morbidity and mortality in this vulnerable group and inform future health policy planning.

In the field of transplant medicine, we observed a lower kidney transplantation rate in CKD patients with HF compared with those without HF (1.0% vs. 1.8%). This difference may reflect selection during transplant evaluation given cardiovascular comorbidity. Based on our data, this indicates a need for structured transplant assessment pathways for CKD–HF candidates and for studies addressing post-transplant outcomes and optimization of pre-transplant HF management in this high-risk group.

### 4.1. Strengths and Limitations

The major strengths of our study include its large sample size, the combination of nationwide administrative claims data with detailed EMR data, and the integration of time-to-event analyses. These features allowed us to assess prevalence, comorbidities, outcomes, and mortality with high statistical power and provide a comprehensive view of HF in CKD patients in Germany. Furthermore, the addition of echocardiographic and laboratory parameters from the EMR cohort offered phenotypic depth beyond administrative coding.

However, several limitations must be acknowledged. The reliance on ICD/OPS coding in claims data introduces the risk of miscoding and underreporting, which may have led to undercoding of HF. In the EMR cohort, impaired LVEF in 7.4% of CKD-no-HF patients supports this possibility. NT-proBNP values were missing in the majority of cases, restricting phenotypic characterization. Follow-up analyses were limited to readmissions within the hospital network, which may underestimate true event rates. Furthermore, differences in CKD definitions between claims (ICD/OPS) and EMR (eGFR-based) cohorts reduce direct comparability. These limitations restrict the generalizability of our findings and should be considered when interpreting the results.

We could not account for socioeconomic status, race/ethnicity, tobacco use, or alcohol consumption, which are relevant modifiers of CKD/HF risk [32,33,34,35,36].

### 4.2. Value and Main Findings

With CaReMe-CKD-HF, we conducted the largest epidemiological study of HF in CKD specifically within a German hospitalized population. We confirmed a high prevalence of HF in CKD, demonstrated increased and earlier mortality, and described elevated emergency readmission rates. We added underreported information on time-to-event outcomes, causes of death, AKI distribution, and the differential prevalence of CKD stages between HF and non-HF patients. By combining nationwide claims data with detailed EMR analyses, we were able to provide a more comprehensive picture, including laboratory and echocardiographic findings. We also identified disparities in transplantation rates between CKD patients with and without HF, highlighting an area for future research. Collectively, these findings underline the medical and socioeconomic importance of HF in CKD and offer a robust basis for healthcare planning, clinical management strategies, and interventional studies.

## 5. Conclusions

This nationwide study shows that nearly half of hospitalized CKD patients in Germany are affected by HF, underscoring the high clinical and public health burden of this comorbidity. The coexistence of CKD and HF calls for systematic risk stratification, earlier detection, and integrated nephrology–cardiology management to mitigate adverse outcomes. Evidence-based therapies, including SGLT2 inhibitors, should be prioritized to improve survival and reduce readmissions in this vulnerable population.

Our findings also have implications for healthcare planning and policy, highlighting the need for adequate resource allocation and structured preventive measures. Prospective and interventional studies are warranted to clarify causal pathways, optimize treatment strategies, and evaluate long-term outcomes, including in the transplant setting.

These conclusions must be interpreted in the context of methodological limitations, particularly reliance on administrative coding and the restriction of follow-up to the hospital network. Nevertheless, this analysis provides robust real-world evidence that can inform both clinical practice and healthcare policy in Germany.

## Figures and Tables

**Table 1:** Baseline characteristics and comorbidities of hospitalized patients with chronic kidney disease, stratified by the presence of heart failure. Age is presented as mean ± standard deviation; categorical variables are presented as % (*n*). *p*-value * indicates the statistical test used: Student’s *t*-test for continuous variables and Fisher’s exact test for categorical variables. Statistically significant *p*-values (*p* < 0.05) are shown in bold.

	Overall Cohort % (*n*)	CKD-HF % (*n*)	CKD-No-HF % (*n*)	*p*-Value *
**Number of patients**	48,011	22,072	25,939	
**Age (years)** **± SD**	73.8 ± 13.1	76.5 ± 10.8	71.5 ± 14.3	**<0.001**
**Female sex**	44% (21,107)	42.9% (9473)	44.9% (11,634)	**<0.001**
**CKD–Chronic**	94.3% (45,256)	94.6% (20,879)	94% (24,377)	**0.004**
CKD stage 1	0.3% (163)	0.3% (75)	0.3% (88)	1
CKD stage 2	6.4% (3074)	6% (1328)	6.7% (1746)	**0.001**
CKD stage 3	29.4% (14,128)	32.2% (7102)	27.1% (7026)	**<0.001**
CKD stage 4	20.4% (9778)	23.7% (5238)	17.5% (4540)	**<0.001**
CKD stage 5	37.6% (18,073)	32.3% (7129)	42.2% (10,944)	**<0.001**
CKD stage unspecified	0.1% (40)	0% (7)	0.1% (33)	**<0.001**
**AKI**	56.4% (27,086)	66.6% (14,690)	47.8% (12,396)	**<0.001**
**ESRD**	50.2% (24,108)	51.2% (11,310)	49.3% (12,798)	**<0.001**
**Dialysis**	41.3% (19,852)	44.2% (9761)	38.9% (10,091)	**<0.001**
**Kidney transplant**	1.4% (693)	1% (217)	1.8% (476)	**<0.001**
**Coronary ischemic disease**	29.4% (14,133)	41.3% (9122)	19.4% (5021)	**<0.001**
**Myocardial infarction**	9.2% (4437)	14% (3097)	5.2% (1340)	**<0.001**
**Atrial fibrillation/atrial flutter**	32.4% (15,541)	46.3% (10,215)	20.5% (5326)	**<0.001**
**Bradycardia/conduction disorder**	5.6% (2668)	8.2% (1805)	3.3% (863)	**<0.001**
**Hypertension**	71.8% (34,471)	77.3% (17,066)	67.1% (17,405)	**<0.001**
**Stroke**	6.6% (3147)	7.4% (1637)	5.8% (1510)	**<0.001**
**Peripheral artery disease**	10.8% (5198)	13.4% (2964)	8.6% (2234)	**<0.001**
**Diabetes mellitus type 2**	47.9% (22,994)	54.1% (11,941)	42.6% (11,053)	**<0.001**
**Major organ specific bleeding**	10.3% (4961)	13.6% (2995)	7.6% (1966)	**<0.001**
**COPD**	10.6% (5083)	14.6% (3220)	7.2% (1863)	**<0.001**
**Cancer**	7.6% (3667)	5.8% (1288)	9.2% (2379)	**<0.001**

Abbreviations: AKI—acute kidney injury; CKD—chronic kidney disease; COPD—chronic obstructive pulmonary disease; ESRD—end-stage renal disease; HF—heart failure; SD—standard deviation.

**Table 2:** Clinical and laboratory parameters from the EMR cohort, stratified by heart failure status. Continuous variables are presented as mean ± standard deviation; categorical variables are presented as % (*n*). *p*-value * indicates the statistical test used: Student’s *t*-test for continuous variables and Fisher’s exact test for categorical variables. Statistically significant *p*-values (*p* < 0.05) are shown in bold.

	Overall Cohort	CKD-HF	CKD-No-HF	*p*-Value *
**Baseline characteristics**				
**Number of patients**	23,377	16,124	7253	
**Age (years)** ** ± SD **	74.5 ± 9.9	74.6 ± 9.9	74.1 ± 9.9	**<0.001**
**Female sex (*n*)**	39.2% (9170)	37.4% (6035)	43.2% (3135)	**<0.001**
**eGFR (mL/min/BSA)** ** ± SD **	52.4 ± 19.5	50.4 ± 19.4	56.9 ± 19.2	**<0.001**
**Measured LVEF (%)** ** ± SD **	49 ± 14.9	44.9 ± 15	60.1 ± 6.9	**<0.001**
**Weight (kg)** ** ± SD **	83.5 ± 18.5	83.9 ± 18.9	82.8 ± 17.8	**<0.001**
**BMI** ** ± SD **	29.1 ± 5.7	29.2 ± 5.9	28.9 ± 5.4	**<0.001**
**SBP (mmHg)** ** ± SD **	142.1 ± 27.3	138.8 ± 27.8	149.4 ± 24.9	**<0.001**
**NT-proBNP (ng/dL)** ** ± SD **	6861.7 ± 16,364.2	8268.3 ± 18,197.2	2182.4 ± 5351.4	**<0.001**
**Hemoglobin (g/dL)** ** ± SD **	7.9 ± 1.3	7.9 ± 1.4	8.1 ± 1.4	**<0.001**

Abbreviations: BMI—body mass index; CKD—chronic kidney disease; eGFR—estimated glomerular filtration rate (mL/min/body-surface-area); HF—heart failure; LVEF—left ventricular ejection fraction; NT-proBNP—N-terminal pro-brain natriuretic peptide; SBP—systolic blood pressure; SD—standard deviation.

**Table 3:** In-hospital mortality of patients with chronic kidney disease (CKD) with and without heart failure (HF) in the claims and EMR cohorts. Values are presented as % (*n*). Odds ratios (ORs) with corresponding 95% confidence intervals (CIs) are displayed. *p*-value * indicates the statistical test used: Fisher’s exact test for categorical variables. Statistically significant *p*-values (*p* < 0.05) are shown in bold.

Claims Data	Overall Cohort % (*n*)	CKD-HF % (*n*)	CKD-No-HF % (*n*)	OR (95% CI)	*p*-Value *
**In-hospital mortality**	11.2% (5395)	16.5% (3650)	6.7% (1745)	2.747 (2.586; 2.919)	**<0.001**
**Renal death**	7.1% (3423)	10.4% (2289)	4.4% (1137)	2.520 (2.340; 2.715)	**<0.001**
**Cardiovascular death**	1.9% (895)	3.5% (765)	0.5% (130)	7.127 (5.905; 8.661)	**<0.001**
**EMR Data**	**Overall Cohort % (*n*)**	**CKD-HF** **% (*n*)**	**CKD-no-HF** **% (*n*)**	**OR** **(95% CI)**	***p*** -Value *
**In-hospital mortality**	4% (946)	5.4% (871)	1% (75)	5.465 (4.305; 7.028)	**<0.001**
**Renal death**	0% (5)	0% (4)	0% (1)	1.799 (0.178; 88.600)	0.999
**Cardiovascular death**	3.5% (815)	4.7% (763)	0.7% (52)	6.879 (5.182; 9.306)	**<0.001**

Abbreviations: CI—confidence interval; CKD—chronic kidney disease; EMR—electronic medical records; HF—heart failure; OR—odds ratio.

**Table 4:** Readmission statistics for the overall chronic kidney disease (CKD) cohort and stratified by heart failure (HF) status. Values are presented as % (*n*). Odds ratios (ORs) with corresponding 95% confidence intervals (CIs) are provided. *p*-value * indicates the statistical test used: Fisher’s exact test for categorical variables. Statistically significant *p*-values (*p* < 0.05) are shown in bold.

Readmission Cause	Overall Cohort (%)	CKD-HF (%)	CKD-No-HF (%)	OR * (95% CI)	*p*-Value *
**Any readmission**					
Any type of readmission	50.1%	49.1%	50.9%	0.931 (0.898; 0.965)	<0.001
Emergency readmission	37.1%	38.2%	36.2%	1.088 (1.048; 1.130)	<0.001
**ESRD or dialysis**					
Any type of readmission	18.8%	17%	20.3%	0.802 (0.766; 0.841)	<0.001
Emergency readmission	11.8%	11.6%	12.1%	0.951 (0.899; 1.006)	0.079
**Kidney disease**					
Any type of readmission	12.4%	12.1%	12.7%	0.943 (0.892; 0.996)	0.034
Emergency readmission	6.7%	7.1%	6.4%	1.122 (1.044; 1.206)	0.002
**Heart failure**					
Any type of readmission	9.7%	14.4%	5.7%	2.808 (2.631; 2.998)	<0.001
Emergency readmission	7.4%	11%	4.4%	2.667 (2.478; 2.870)	<0.001
**Myocardial infarction**					
Any type of readmission	1.5%	1.7%	1.4%	1.236 (1.064; 1.436)	0.005
Emergency readmission	1.3%	1.4%	1.1%	1.230 (1.044; 1.450)	0.012
**All cause In-hospital death**					
Any type of readmission	10.8%	13.3%	8.7%	1.609 (1.518; 1.706)	<0.001
Emergency readmission	8.3%	10.4%	6.5%	1.655 (1.549; 1.768)	<0.001
**Cardiovascular death**					
Any type of readmission	2.9%	4.2%	1.7%	2.520 (2.245; 2.833)	<0.001
Emergency readmission	2.1%	3.2%	1.3%	2.549 (2.231; 2.918)	<0.001
**Renal death**					
Any kind of readmission	1.2%	1.6%	0.9%	1.872 (1.580; 2.221)	<0.001
Emergency readmission	0.9%	1.3%	0.6%	2.038 (1.676; 2.485)	<0.001

Abbreviations: CI—confidence interval; CKD—chronic kidney disease; ESRD—end-stage renal disease; HF—heart failure; OR—odds ratio.

**Table 5:** Time-to-first readmission analysis for the overall chronic kidney disease (CKD) cohort and stratified by heart failure (HF) status. Continuous variables are presented as mean ± standard deviation. *p*-value * indicates the statistical test used: Student’s *t*-test for continuous variables. Statistically significant *p*-values (*p* < 0.05) are shown in bold.

Readmission Cause	Overall CKD Cohort Days ± SD	CKD-HF Days ± SD	CKD-No-HF Days ± SD	*p*-Value *
**Any readmission**	196.2 ± 291	175.9 ± 266.1	212.8 ± 309	**<0.001**
**HF**	336.1 ± 400.6	290.3 ± 360.1	435.2 ± 461.5	**<0.001**
**AMI**	468.8 ± 453	437.2 ± 448.9	501.9 ± 455.7	0.056
**Hyperkalemia**	365.8 ± 371.4	334.7 ± 377.8	394.6 ± 429.6	**<0.001**
**ESRD or dialysis**	226.3 ± 319.3	212.7 ± 301.5	236 ± 331.1	**<0.001**
**Stroke**	506.2 ± 470	507.8 ± 464.6	504.8 ± 475.4	0.931
**All-cause in-hospital death**	439 ± 447.5	414.3 ± 446.8	471 ± 492.4	**<0.001**
**CV death**	436.7 ± 457	414.7 ± 440.5	482.7 ± 482	**0.013**

Abbreviations: AMI—acute myocardial infarction; CKD—chronic kidney disease; CV—cardiovascular; ESRD—end-stage renal disease; HF—heart failure.

## Data Availability

The datasets generated and/or analyzed during the current study are not publicly available due to data protection regulations, but are available from the corresponding author on reasonable request.

## Supplementary Materials

The following supporting information can be downloaded at: https://www.mdpi.com/article/10.3390/jcdd12110448/s1, Table S1: ICD-10 and OPS codes for index cohort characterization and HF; Table S2: ICD-10 and OPS codes for CKD stages and comorbidities; Table S3: Missing values (EMR); Table S4: STROBE statement checklist.

## Abbreviations

The following abbreviations are used in this manuscript:

AKI	acute kidney injury
CaReMe	observational study initiative to highlight the epidemiology and healthcare burden of CArdiovascular-REnal-MEtabolic diseases
CI	confidence interval
CKD	chronic kidney disease
CKD-EPI	Chronic Kidney Disease Epidemiology Collaboration
CKD-HF	CKD patients with comorbid HF
CKD-no-HF	CKD patients without comorbid HF
Claims data	routine administrative data on diagnoses and procedures
CVD	cardiovascular disease
eGFR	estimated glomerular filtration rate
ESRD	end-stage renal disease
EMR	electronic medical records
FU	follow-up
HF	heart failure
HFmrEF	heart failure with mildly reduced ejection fraction
HFpEF	heart failure with preserved ejection fraction
HFrEF	heart failure with reduced ejection fraction
ICD-10-GM	International Statistical Classification of Diseases and Related Health Problems (German Modification)
LVEF	left ventricular ejection fraction
NT-proBNP	n-terminal prohormone of brain natriuretic peptide
OPS	Operations and Procedures (German adaptation of the International Classification of the Procedures in Medicine of the World Health Organization, version 2017)
OR	odds ratio
py	patient–years
