# Supplementary material for: CaReMe-CKD-HF–Epidemiology of Heart Failure in Chronic Kidney Disease: A Retrospective Analysis of Routine Administrative Data from a German Hospital Network
Source: J Cardiovasc Dev Dis. 2025 Nov 19;12(11):448. doi: 10.3390/jcdd12110448 (PMC12653909; doi:10.3390/jcdd12110448)
Supplement: Supplementary file 1 [file jcdd-12-00448-s001.zip › CaReMe_CKD_HF_Supplemental Table S1.pdf]

Supplemental Table S1: ICD-10 and OPS codes for index cohort characterization and HF stratification

	ICD-10 code	OPS code
**Chronic kidney disease**	N03; N18; N19; I12.0; I13.1; I13.2; N08.3; E11.2; E12.2; E13.2; E14.2; Z49.1; Z49.2; Z99.2	8-853.0; 8-853.3; 8-853.4; 8-853.5; 8-853.6; 8-854.0; 8-854.2; 8-854.3; 8-854.4; 8-854.5; 8-854.x; 8-854.y; 8-855.0; 8-855.3; 8-855.4; 8-855.5; 8-855.6; 8-855.x; 8-855.y; 8-857; 8-85a
**Acute kidney injury**	N17	
**Heart failure**	I11.00; I11.01; I13.01; I13.20; I13.21; I25.5; I42.0; I42.6; I42.6; I42.7; I50	

Cohort definition as for claims: patients with one of the following conditions:

- Main discharge diagnosis CKD
- Main discharge diagnosis Acute kidney injury AND secondary discharge diagnosis CKD
- (Main OR secondary discharge diagnosis CKD) AND at least one CKD OPS code within index case)

Cohort definition as for EMR: patients with one of the following conditions:

- Main OR secondary diagnosis CKD
- CKD associated OPS code
