# Supplementary material for: CaReMe-CKD-HF–Epidemiology of Heart Failure in Chronic Kidney Disease: A Retrospective Analysis of Routine Administrative Data from a German Hospital Network
Source: J Cardiovasc Dev Dis. 2025 Nov 19;12(11):448. doi: 10.3390/jcdd12110448 (PMC12653909; doi:10.3390/jcdd12110448)
Supplement: Supplementary file 1 [file jcdd-12-00448-s001.zip › CaReMe_CKD_HF_Supplemental Table S2.pdf]

Supplemental Table S2: ICD-10 and OPS codes for CKD stages and comorbidities

	ICD-10 code	OPS code
**CKD**		
**CKD stage 1**	N18.1	
**CKD stage 2**	N18.2	
**CKD stage 3**	N18.3	
**CKD stage 4**	N18.4	
**CKD stage 5**	N18.5	
**CKD stage unspecified**	N18.8; N18.9	
**CKD unspecified**	N18.8; N18.9; N19	
**Comorbidities**		
**Coronary ischemic disease**	I21; I22; I23.0; I.231; I23.2; I23.3; I23.4; I23.5; I23.6; I23.8; I25.0; I25.11; I25.12; I25.13; I25.14; I25.15; I25.16; I25.17; I25.18; I25.19; I25.2; I25.3; I25.4; I25.5; I25.6; I25.8; I25.9	
**Myocardial infarction**	I21; I22; I25.2	
**Cardiovascular revascularization**		
**CABG**		5-361; 5-362; 5-363; 5-364
**PCI with stent**		8-837.3; 8-837.k; 8-837.m; 8-837.n; 8-837.p; 8-837.u; 8-837.v; 8-837.w
**PCI without stent**		8-837.0; 8-837.1; 8-837.2; 8-837.5; 8-837.6; 8-837.q; 8-837.t
**Unstable angina**	I20.0	
**Angina pectoris**	I20.1; I20.8; I20.9	
**Atrial fibrillation / atrial flutter**	I48	
**Bradycardia / conduction disorder**	I44; I45.0; I45.1; I45.2; I45.3; I45.4; I45.5; I45.9; I49.5; R00.1	

**Supraventricular arrhythmia (other than atrial fibrillation / atrial flutter)**	I45.6; I47.1	
**Ventricular arrhythmia**	I47.0; I47.2; I49.0; I49.3	
**Hypertension**	I10; I11; I12; I13; I15	
**Hypotension**	I95	
**Stroke**	G45; I61; I63; I64; I69.1; I69.2; I69.3; I69.4; I69.8	
**Hemorrhagic**	I61	
**Ischemic**	I63	
**Transitory ischemic attack**	G45	
**Peripheral artery disease**	I70.2; I73.9; I74.2; I74.3; I74.4; I74.5; I74.6; I74.7; I74.8; I74.9	
**Lower limb amputation**	Z89.4; Z89.5; Z89.6; Z89.7; Z89.8	5-864; 5-865; 5-866.3; 5-866.4; 5-866.5
**CKD - Diabetic**	E11.2; E12.2; E13.2; E14.2; N08.3	
**CKD - Hypertensive**	I12.0; I13.1; I13.2	
**CKD - Glomerular disease**	N00; N01; N02; N03; N04; N05; N06; N07; N08	
**CKD - Renal tubulo-interstitial disease**	N10; N11; N12; N13; N14; N15; N16	
**CKD - Dialysis**	T82.4; T85.71; Z49.1; Z49.2; Z99.2	8-853.0; 8-853.3; 8-853.4; 8-853.5; 8-853.6; 8-854.0; 8-854.2; 8-854.3; 8-854.4; 8-854.5; 8-854.x; 8-854.y; 8-855.0; 8-855.3; 8-855.4; 8-855.5; 8-855.6; 8-855.x; 8-855.y; 8-857; 8-85a
**End stage renal disease**	N18.5; T82.4; T85.71; Z49.1; Z49.2; Z99.2	8-853.0; 8-853.3; 8-853.4; 8-853.5; 8-853.6; 8-854.0; 8-854.2; 8-854.3; 8-854.4; 8-854.5; 8-854.x; 8-854.y; 8-

		855.0; 8-855.3; 8-855.4; 8-855.5; 8-855.6; 8-855.x; 8-855.y; 8-857; 8-85a
**Kidney transplant (listed)**	Z75.60; Z75.64; Z75.70; Z75.74	1-920.00; 1.920.10; 1-920.20; 1-920.30; 8-979.20; 8-97c.00; 8-97c.20; 8-97c.40; 8-97c.50
**Kidney transplant**	T86.1; Z94.0	5-555
**Diabetes mellitus type 2**	E11; E12; E13; E14	
**Major organ specific bleeding**	D62; I60; I61; I62; I85.0; K22.6; K25.0; K25.2; K25.4; K25.6; K26.0; K26.2; K26.4; K26.6; K27.0; K27.2; K27.4; K27.6; K28.0; K28.2; K28.4; K28.6; K29.0; K62.5; K92.0; K92.1; K92.2	
**Bariatric surgery**		5-445.4; 5-445.5; 5-448.a; 5-448.b; 5-448.c; 5-448.d; 5-448.e; 5-448.f; 5-449.2; 5-449.h; 5-449.k; 5-449.n; 5-449.p; 5-449.s
**Diabetic mono-/polyneuropathy**	E11.4; E12.4; E13.4; E14.4; G59.0; G63.2	
**Diabetic eye complications**	E11.3; E12.3; E13.3; E14.3; H36.0	
**Diabetic foot/Peripheral angiopathy**	E11.5; E12.5; E13.5; E14.5; M14.2	
**Diabetic kidney disease**	E11.2; E12.2; E13.2; E14.2; N08.3	
**Diabetes with several-/unspecified complications**	E11.6; E12.6; E13.6; E14.6; E11.7; E12.7; E13.7; E14.7; E11.8; E12.8; E13.8; E14.8	
**Severe hypoglycemia**	E11.0; E12.0; E13.0; E14.0; E16.0; E16.1; E16.2	

**Keto-/lactate acidosis**	E11.1; E12.1; E13.1; E14.1	
**Cancer**	C0; C1; C2; C3; C4; C5; C6; C7; C8; C9	
**COPD**	J44	
**Liver disease**	B18; I98.2; I98.3; K70; K71.1; K71.3; K71.4; K71.5; K71.7; K72; K73; K74; K76.0; K76.2; K76.9; Z94.4	
**Liver transplant**	T86.4; Z94.4	5-504
**Gout**	M10; M14.0	
**Hyperlipidemia**	E78	
**AIDS / HIV infection**	B20; B21; B22; B23; B24	

CKD: chronic kidney disease; CABG: coronary artery bypass graft; PCI: percutaneous coronary intervention; COPD: chronic obstructive pulmonary disease; AIDS: acquired immune deficiency syndrome; HIV: human immunodeficiency virus
