# Supplementary material for: CaReMe-CKD-HF–Epidemiology of Heart Failure in Chronic Kidney Disease: A Retrospective Analysis of Routine Administrative Data from a German Hospital Network
Source: J Cardiovasc Dev Dis. 2025 Nov 19;12(11):448. doi: 10.3390/jcdd12110448 (PMC12653909; doi:10.3390/jcdd12110448)
Supplement: Supplementary file 1 [file jcdd-12-00448-s001.zip › CaReMe_CKD_HF_Supplemental Table S3.pdf]

Supplemental Table S3 – Missing values (EMR)

Variable	Missing values in total	CKD-no-HF	CKD-HF
**Age**	0 (0.0%)	0 (0.0%)	0 (0.0%)
**Height**	3156 (13.5%)	864 (11.9%)	2292 (14.2%)
**Weight**	3357 (14.4%)	921 (12.7%)	2436 (15.1%)
**BMI**	3357 (14.4%)	921 (12.7%)	2436 (15.1%)
**LVEF (%)**	5142 (22.0%)	2271 (31.3%)	2871 (17.8%)
**LVEF category**	4642 (19.9%)	2106 (29.0%)	2536 (15.7%)
**NT-ProBNP (ng/l)**	18562 (79.4%)	6139 (84.6%)	12423 (77.0%)
**Creatinine (mg/dl)**	80 (0.3%)	33 (0.5%)	47 (0.3%)
**eGFR (ml/min/BSA)**	139 (0.6%)	52 (0.7%)	87 (0.5%)
**HbA1c (%)**	18433 (78.8%)	5957 (82.1%)	12476 (77.4%)
**Hemoglobin (g/dl)**	113 (0.5%)	45 (0.6%)	68 (0.4%)
**Hematocrit (%)**	113 (0.5%)	45 (0.6%)	68 (0.4%)
**Potassium (mmol/l)**	440 (1.9%)	130 (1.8%)	310 (1.9%)
**Uric acid (mg/dl)**	21298 (91.1%)	6842 (94.3%)	14456 (89.6%)
**Troponin T hs (ng/l)**	9761 (41.7%)	3574 (49.3%)	6187 (38.4%)
**Systolic blood pressure**	947 (4.1%)	208 (2.9%)	739 (4.6%)
**Medication**	2213 (9.9%)	577 (8.0%)	1636 (10.7%)
